# How Health Professionals Conceptualize and Represent Placebo Treatment in Clinical Trials and How Their Patients Understand It: Impact on Validity of Informed Consent

**DOI:** 10.1371/journal.pone.0155940

**Published:** 2016-05-19

**Authors:** Pascal-Henri Keller, Olivier Grondin, François Tison, Francois Gonon

**Affiliations:** 1 Department of Psychology, University of Poitiers, 86000, Poitiers, France; 2 Department of Psychology, University of Bordeaux, 33076 Bordeaux, France; 3 Institute of Neurodegenerative Diseases, University of Bordeaux, 33076 Bordeaux, France; 4 Centre National de la Recherche Scientifique, CNRS UMR5293, 33076 Bordeaux, France; Cardiff University, UNITED KINGDOM

## Abstract

**Context:**

Previous studies suggested that many patients, who have given their informed consent to participate in randomized controlled trials (RCT), have somewhat limited understanding of what a placebo treatment is. We hypothesized that the relationship between patients and their health professionals plays a central role in this understanding.

**Methods:**

We interviewed 12 patients included in RCTs (nine suffering from Parkinson’s disease and three from Huntington’s disease) and 18 health professionals involved with RCTs (eight principal investigators, four associated physicians and six clinical research associates). Semi-structured interviews were conducted after the RCTs had ended but before the treatment allocation was revealed.

**Results:**

Only two patients clearly understood the aim of placebo-controlled RCTs. Only one principal investigator said she asks all her patients whether they agree to participate in RCTs. The seven others said they only ask patients who seem more likely to be compliant. Their selection criteria included docility and personality traits associated in other studies with enhanced placebo responses. According to 13 of the 18 health professionals, their relationship with patients may influence the amplitude of the placebo response. All but one clinical research associates added that the placebo response could result from a “maternal” type of care. All principal investigators said they have a strong influence on their patient's decision to participate. Finally, when interviewees were asked to narrate a memory of a medically unexplained healing, in eight of 11 physicians' narratives the beneficiary was a child while in 10 of 12 patients' narratives it was an adult.

**Conclusion:**

Our observations suggest that the interrelationship between health professionals and patients involved in RCTs could be compared to that between parents and children. Therefore, adherence to formal rules regarding informed consent does not ensure a balanced relationship between patients and health professionals.

## Introduction

Double-blinded randomized controlled trials (RCTs) are mandatory for assessing the effectiveness of new treatments. When an effective therapy has been proven to improve survival or to decrease morbidity in the context of serious illness, it must be used as a control treatment [1]. However, placebo treatment is still extensively used in the development of new pharmaceuticals either when there is no proven therapy or to assess the sensitivity of the trial [1]. Indeed, the effect size of a placebo treatment is highly variable depending on the pathology [2], the psychosocial context and individual factors related to patients and health professionals [3, 4]. Thus, because the effect size of the active treatment for many medical conditions is only mildly superior to that of a placebo treatment [5], a placebo arm is required in most RCTs to assess the sensitivity of the essay and the effectiveness of the treatment under investigation [1].

A placebo treatment involves the administration of either a sham treatment or an inactive pharmaceutical, e.g. a sugar pill. However, Moerman emphasized (p. 130) that patients do not respond to placebo: "they respond to language, to caring, to culture, to community, to history" [6]. A "placebo response" is an intrinsic component of almost all medical treatments. Indeed, the open administration of a medication is significantly more effective than its hidden administration [3, 4]. Along the same lines, Blease (2012) proposed that, in clinical practice, the term “placebo effect” should be replaced with “positive care effect” [7]. The present study, however, focuses on placebo treatment in RCTs and, thus, we use the standard term “placebo response” throughout the article although we agree with Blease and Moerman that this term is inadequate.

Numerous studies have investigated the psychosocial components of the placebo response. The most frequently cited include expectation, conditioning to medical environment and interpersonal relationship between patients and health professionals [3, 4, 8]. The expectation component has been revealed by experiments modulating the probability of receiving either a placebo or a treatment said to be effective, whereas all of the subjects actually received the same treatment. Such studies have been performed either with a placebo or with an active drug, in healthy volunteers or in the context of various pathological conditions including Parkinson's disease. They have consistently shown that clinical outcomes are positively related to the expected probability of receiving a supposedly active treatment [4, 9–11]. Other studies have successfully disentangled the interpersonal relationship component from the effects of conditioning by the medical ritual [8]. According to a recent meta-analysis, the patient-clinician relationship has a small but statistically significant effect on health outcomes [12].

Although the placebo response appears as a robust phenomenon at a population level, its appearance is virtually unpredictable at the level of individual patients. Indeed, its stability over time in individual subjects has not been clearly established [12]. Moreover, until recently, studies investigating the psychological profile of placebo responders failed to produce any strong or consistent findings [13]. Nevertheless, a few recent studies suggest that some personality traits are associated with a larger placebo response, namely dispositional optimism [14–16], extraversion and agreeableness [17, 18]. However, these and other studies reviewed by Jaksic et al. (2013) and Horing et al. (2014) showed that the moderating effects of personality on placebo response also depend on the situation [13, 19]. In particular, optimism and extraversion are only associated with larger placebo responses in situations that include warm emphatic interactions with caregivers, which presumably promote a positive expectancy.

Patients' cognitive and emotional representations of RCTs and of placebo treatment have already been investigated because they may influence the willingness of patients to participate in RCTs [20]. Moreover, inaccurate lay interpretation of RCT concepts may undermine the validity of the informed consent given by RCT participants [21]. Bishop et al. (2012) reviewed the studies investigating how RCT participants conceptualize placebo and concluded (p.768): "Existing research suggests that lay people have somewhat limited understanding of placebos and their effects". Their own observations are consistent with these previous studies. They interviewed 12 patients assigned to the placebo arm of an RCT and observed that only three understood its scientific necessity [21].

Cognitive and emotional representations of the placebo phenomenon have been less explored among health professionals than among patients. Several authors have conceptualized and described the conflicts that trial staff experience between their clinical and research roles [22–25]. In particular participant recruitment is a major challenge to RCTs [24, 26, 27]. RCTs bring into play interpersonal relationships that are much more complex than those occurring in the more ordinary context of a one-to-one dialogue between a physician and an outpatient. Indeed, at least three types of health professionals are involved in most RCTs: the principal investigator (PI), who is always a medical doctor, the associated physician (AP) who is in charge of the follow-up of individual patients, and the clinical research associate (CRA) who is rarely a medical doctor. Since all interact with RCT participants, their own representation of the placebo response might affect patients’ representations. Therefore, patients and health professionals' representations of the placebo treatment deserve more in-depth investigations. Using semi-structured interviews, the present study investigated the representations of the placebo phenomenon among eight PIs, four APs and six CRAs, as well as 12 patients recruited in RCTs by these PIs. We investigated placebo representations in the context of two irreversible neurodegenerative pathologies with no proven protective treatment, Parkinson's and Huntington's diseases.

## Methods

Patients were interviewed because they were previously involved in placebo-controlled RCTs related to drug treatments. They lived in two areas in Western France (Bordeaux and Angers). This research was approved on September 26th, 2012 by the local bioethics committee (CPP SOOM2, Bordeaux) in agreement with French law (Hurriet-Sérusclat Law of December 20, 1988, subsection IIa article 15). Because interviews with patients had no therapeutic aim, the committee considered that an oral consent from interviewees sufficed. All persons who were asked about a possible interview agreed to participate and expressed their oral consent to the interviewer. Semi-structured face-to-face interviews were conducted between October 2012 and March 2014 by the same author (PHK). Health professionals were interviewed in four neurology departments, three in France (Paris, Angers, Bordeaux) and one in Switzerland (Geneva). RCT consent procedures were similar in all departments: the patients met the PI, who informed them about the RCT and asked them whether they were willing to participate. However, patients’ written consent to participate was managed and received by the corresponding CRA after the initial consultation with the PI.

The interviewer (PHK) had no relationship with the interviewees prior to study commencement or after their interview. One of the present authors (FT) recruited all eight PIs. Among them, two PIs (one in Bordeaux and one in Angers) recruited for interviews 12 patients, their four corresponding APs and five CRAs. The sixth CRA was recruited in Paris by a third PI. None of the six CRAs was a physician. Therefore, the interviewer and the authors involved in the content analysis reported ahead (PHK, FG and OG) did not take part in the recruitment process.

Most interviewees were concerned with Parkinson's disease and only a few with Huntington's disease (Table 1). However, two PIs were dealing with both diseases (Table 1). Patients, APs and CRAs concerned with Parkinson's disease were involved in two RCTs supported by pharmaceutical companies whereas those concerned with Huntington's disease took part in one academic RCT. We observed no obvious differences in the responses of interviewees concerned about either disease, except for side effects, as reported in the Results.

**Table 1:** Interviewees.

Disease	PI (n = 8)	AP (n = 4)	CRA (n = 6)	Patient (n = 12)
Parkinson	6	3	4	9
Huntington	4	1	2	3

AP: associated physician; CRA: clinical research associate; PI: principal investigator

All patients had expressed their consent to participate in their respective RCTs by signing a consent form. Each form for participation in the Parkinson’s disease-related RCT included a statement defining the placebo treatment as “a dummy treatment looking like the real treatment, but without active substance.” The consent form for participation in the Huntington’s disease-related RCT defined a placebo “as a substance that looks like the real treatment, but which is inactive”.

The study was conducted in the context of RCTs that had an inclusion criterion about patients' cognitive capabilities. Therefore, no patient suffered from cognitive deficit at the time of inclusion on the basis of standard tests. Patients were interviewed a few months after these tests and the interviewer (a clinical psychologist) did not notice any signs of cognitive decline.

A total of 12 patients and 18 health professionals were interviewed (Table 1). One AP was interviewed four times about his relationships with four patients and one AP was interviewed twice for the same reason. All patients and the four corresponding APs were met in the context of RCTs that had already ended, but before blinding had been unveiled. Therefore, when interviewed, patients and their close health professionals were not informed of the actual treatment received by the patients. In contrast, the eight PIs and six CRAs were interviewed in a more general context and were not asked about specific patients participating in specific RCTs. All APs and all but one PI were male whereas all six CRAs were female.

Interviewees were met alone and invited to answer several questions specifically related to their role in the RCT (Table 2). Interviews were recorded, fully transcribed and anonymized by the same author (PHK). Their content was then analyzed according to binary or ternary codes that tested whether a specific opinion was stated or not by each interviewee (Tables 3 to 7). Opinions were defined *a posteriori* by two authors (PHK and FG) who also performed the first coding of the interviews. In order to prevent idiosyncratic interpretation, the interviews were then entrusted to a third author (OG) who was not involved in any of the previous steps of the research. This author independently coded the previously defined opinions. The few disagreements between coders were discussed and resolved to establish the classifications reported in the Results section. For each interview, S1 to S12 Tables provide the key sentences upon which every judgment regarding each opinion was based (see Supporting Information).

**Table 2:** List of questions asked to interviewees.

Questions	PI	AP	CRA	patient
	n = 8	n = 4	n = 6	n = 12
1) What do you think about the principle of placebo treatment in RCTs?	x	x	x	x
2) How do you explain the placebo effect?	x	x	x	
3) Usually, how do you describe a placebo-controlled RCT to a patient?	x		x	
4) Do you have personal criteria for recruiting a patient for a placebo-controlled RCT?	x			
5) What’s your involvement in your patient’s decision to participate in an RCT?	x			
6) Do you think you could influence your patient’s response to placebo?	x		x	
7) Do you think you could influence the treatment response of your patient?		x		
8) Do you think your physician could influence your treatment response?				x
9) Do you think physicians could influence placebo responses?			x	
10) Could you remember a story about healing unexplained in medicine?	x	x	x	x

AP: associated physician; CRA: clinical research associate; PI: principal investigator

**Table 3:** Conceptualization of the placebo response.

Opinions expressed in response to questions 1 and 2		PI	AP	CRA	patient
		n = 8	n = 4	n = 6	n = 12
a) In RCTs, placebo is a methodological requirement to assert the effectiveness of the new treatment under investigation.		8	4	6	2
b) Mutually exclusive opinions	Neurobiological processes are involved.	2	1	0	NR
	Expectations induce neurobiological effects.	6	2	1	NR
	Placebo treatment induces expectations and beliefs.	0	1	5	NR
c) The interrelationship with health professionals is involved.		6	3	4	NR
d) Patients allocated to placebo might feel disappointed.		0	1	4	2

AP: associated physician; CRA: clinical research associate; PI: principal investigator; NR = not relevant

**Table 4:** Opinion of principal investigators about patients' inclusion in RCTs.

Opinions expressed in answers to questions 4 and 5	n = 8
a) The PI has subjective criteria for including patients.	7
b) The PI also considers the patient’s family circle.	4
c) The PI acknowledges that he influences the patient’s decision.	8

PI: principal investigator

**Table 5:** General influence of PI and CRA on placebo response.

Opinions expressed in answers to question 6: "Do you think you could influence the patient’s response to placebo?"		PI	CRA
		n = 8	n = 6
a) Do you think you have an influence on the placebo response?		Yes: 6	Yes: 3
		Maybe: 2	Maybe: 3
		No: 0	No: 0
b) How it works.	Through my enthusiasm and my power of persuasion.	6	
	It results from the care and support provided by our department.	2	
	It results from a maternal-type of care and support.		5
	It works through suggestion.		1

CRA: clinical research associate; PI: principal investigator

**Table 6:** Specific influence of APs on treatment response of their patients.

Opinions expressed in answers to questions 7,8, 9	AP	patient	CRA
	n = 4	n = 12	n = 6
a) As an AP, I think I had an influence on the treatment response of my patients.	agree: 0		
	disagree: 2		
	DK: 2		
b) I think my physician (i.e. my AP) had an influence on my treatment response.		agree: 1	
		disagree: 9	
		DK: 2	
c) I think APs might influence the placebo response.			agree: 3
			disagree: 0
			DK: 3

AP: associated physician; CRA: clinical research associate; DK: don't know

**Table 7:** Analysis of personal memories of unexplained healings.

Who is said to benefit from the unexplained healing?	PI+AP	CRA	patient
	n = 11	n = 6	n = 12
A child or an adult described as having childlike traits.	8	2	2
An adult.	3	4	10

## Results

### Overview of the interviews

All subjects solicited for an interview accepted to participate and many expressed their interest in the research. Accordingly, none of the participants stopped the interview before the last question. Interviews’ durations ranged from 14 to 48 min (mean ± S.D.: 29.8 ± 9.8). The same questions were asked to all interviewees in each category as indicated in Table 2. When interviewees did not answer or when their answer seemed too vague, the interviewer rephrased the question (see examples ahead). The content analysis of the interviews showed that the answers were more complex than expected. Therefore, two authors (PHK and FG) inferred defined opinions as described in Tables 3 to 7. The presence or absence of any opinion was tested as described in the methods and ascertained by key quotes extracted from each interview as reported in S1 to S12 Tables (see Supporting Information).

### Conceptualization of placebo treatment in RCTs

Opinions regarding the conceptualization of placebo treatment were extracted from the interviewees' answers to the first and second questions (see all quotes in S1 Table). As expected, all health professionals clearly and quickly answered the first question about the placebo arm being required in RCTs to assert the effectiveness of the new treatment under investigation. For example PI-7 stated: *"It is what appears to me today as the soundest method to avoid overestimating the treatment effect*.*"* In contrast, only two out of 12 patients' answers suggested that they had understood why a placebo treatment is required in RCTs, although all of them had signed a consent statement that explained it. Patient P5 stated: *"…to know for sure if the drug is working or not…"* and patient P6 stated: *"…to be able to check whether the drug is active or not*.*"* Two other patients (P2 and P11) vaguely mentioned a need but without expressing an understanding of the usefulness of placebo control. Patient P2 stated: *"It is essential for launching a new drug*.*"* Patient 11 stated: *"It is essential for research studies*, *but for the patient…"* The eight other patients did not talk about placebo as a methodological requirement despite the interviewer's insistence. They only mentioned placebo treatment from their own point of view. For example the interviewer insisted: *"What is the reason for prescribing either a drug or a placebo"*? Patient P1 answered: *"Ultimately*, *I think that I had a placebo because it had no effect*.*"* Moreover, five out of nine patients suffering from Parkinson's disease did not understand that a placebo treatment is a sham treatment with an inactive medication only. For example, one patient said: *"we take a medication with something in it*, *although placebo*, *but there must be something else in it*.*”* The three patients suffering from Huntington disease were more conscious that a placebo medication is inactive because they were told that the active treatment usually induces obvious side effects, including bad breath. Table 3 summarizes the opinions about the conceptualization of the placebo treatment.

Some CRAs also seemed conscious that most patients did not understand what a placebo treatment is. For example one CRA reported her dialogue with a male patient who complained that he perceived no benefit from the treatment. The CRA replied to him that it might be because he received a placebo treatment and the patient answered: *"but*, *the placebo*, *after all*, *it is just like receiving a treatment"*. The CRA did not disabuse him of thinking that the placebo is a real treatment.

Health professionals' answers to the second question were much less clear with many hesitations and inconsistencies (see all quotes in S2 Table). They can be summarized as falling within three distinct opinions explaining the placebo response by means of internal processes in patients. Three health professionals only evoked neurobiological processes. For example AP-2 stated: *"The placebo activates the reward system…the mesocorticolimbic system*.*"* Six others only used psychological descriptions (e.g. expectation, beliefs) and nine described the placebo response as resulting from neurobiological events triggered by expectation. For example, PI-4 stated: *"The one who thinks he received the active molecule… our brain or our psyche is able to secrete a certain number of neurotransmitters*, *hormones…"* Table 3 summarizes the opinions explaining the placebo response. Interestingly, all but one physician evoked neurobiological processes whereas only one CRA did so. In contrast, all but one CRA only used a psychological description of the placebo response. Moreover, 13 of 18 health professionals spontaneously added (see all quotes in S3 Table) that the interrelationship between health professionals and patients might play a role in the placebo response. For example CRA-1 stated: *"Patients get better because their follow-up is more frequent*, *it's real medical management*.*"*

Finally, only two patients spontaneously added that they would feel disappointed if they actually received the placebo treatment. Patient P-3 said: *"If for six months we eats a placebo*, *we will feel more like a guinea-pig than anything else*.*"* Four of the six CRA, but only one physician (an AP), also spontaneously expressed the feeling that it might be disappointing for patients to be allocated to the placebo arm (see all quotes in S4 Table). For example, CRA-6 said: *"It's true that patients don't really like to know they’re only getting the placebo*.*"* In contrast, none of the PI mentioned that patient allocated to placebo arm might feel disappointed (Table 3).

### Patients' inclusion in placebo-controlled RCTs

In the third question PIs and CRAs were asked how they would describe placebo-controlled RCTs to patients. Because answers to this question were conventional, expected and not very informative, we do not systematically comment on them here except for one point. Four CRAs and four PIs said that they usually portray the placebo treatment as an "inactive treatment" or an "inactive molecule". The other PIs (4/8) and CRAs (2/6) did not mention in their answer to the third question how they describe the placebo treatment to patients.

In contrast, PIs' answers to the fourth and fifth questions were internally consistent (see all quotes in S5 Table). Only one PI clearly stated that she asks all of her patients whether they would agree to participate in RCTs. Six PIs said without any hesitation that they avoid asking certain patients. For example PI-3 stated: "*We would not ask patients with a schoolteacher profile*. *These people systematically question what physicians say*.*"* Another said that he does not ask *"anxious patients"*. A third said that he selects patients *"without much personality*.*"* The eighth PI ambiguously answered this question (see quote in S5 Table). All seven PIs put forward criteria for selecting patients with the highest probability of being compliant with the treatment. Half of the PIs spontaneously added (see quotes in S6 Table) that they also consider the family circle of the patient. They select patients with strong family support and avoid those living with a partner who seems critical of the treatment. For example, PI-5 stated: *"The patient needs to have people around him with a positive attitude towards treatment*.*"* In agreement with this selection of patients on the basis of subjective criteria, all PIs acknowledged (see all quotes in S7 Table) that they strongly influenced patients' decisions to participate in an RCT (Table 4). For example PI-5 stated: *"If I set my mind on getting someone to take part*, *he will take part*.*"* Table 4 summarizes the opinions expressed by the PIs about patients' inclusion in RCTs.

### Impact of the interrelationship on the placebo response

The sixth question explored the opinions of PI and CRA about their possible influence on the placebo response as a general phenomenon. Most PIs and CRAs thought that they might have an influence on the placebo response (see all quotes in S8 Table). However, explanations put forward in PIs' and CRAs' answers differed. Most PIs emphasized that their personal belief, hope and enthusiasm might be passed on to patients. For example, PI-1 stated: *"Yes*,*… our enthusiasm*, *our belief in the value of this new drug*, *plays a major role on the patient's involvement… the expectation will be stronger*.*"* In contrast, five out of six CRAs underlined that they took care of their patients in a “maternal” way. For example CRA-1 said: *"Yes*, *we exert a huge influence…It is a little bit like a maternal attitude*, *because as soon as they have a concern*, *they call me*. *Some patients say*: *"We feel pampered*, *like with a mom*.*"* Table 5 summarizes PIs’ and CRAs’ opinions about their influence on the placebo response.

Because we hypothesized that the interrelationship between the four AP and their respective patients might be of particular importance regarding the placebo response, we explored more specifically APs' and patients' opinions through questions 7 to 9. To this end, two APs were interviewed four and two times about their respective patients. The other APs were interviewed only once about their patients. Thus, we asked all four APs about their possible influence on the course of the disease of their eight specific patients. In parallel we asked these eight patients, as well as four additional patients, whether they thought their relationship with their AP contributed to their treatment response. Because no AP said they might have an influence on the course of the disease (see quotes in S9 Table) and because all but one patient denied that their AP might have influenced their treatment response (see quotes in S10 Table), we gave up trying to link patients' opinions with the opinion expressed by their respective APs. Moreover, because the opinions expressed by APs were always the same irrespective of whether their patients got better or not, we give only these general opinions in Table 6. Finally, we also asked CRAs for their general opinion about the possible influence of APs on the treatment response of their patients (see quotes in S11 Table). For example CRA-4 stated: *"Yes*, *some doctors are good listeners and will spend much more time than others*. *It might have an effect*.*"* Comparisons between opinions summarized in Tables 5 and 6 were especially interesting. While most PIs and CRAs believed they have an influence on the placebo response (Table 5), most direct stakeholders, namely APs and patients, denied that the interrelationship between them might influence the placebo response (Table 6). However, half of the CRAs thought that the interrelationship between APs and patients might influence it (Table 6).

### Possible conflicts between research and clinical roles

A growing body of literature has documented that many health professionals involved in RCTs experience conflicts between their research and clinical roles [25]. However, in the present study, none of the 12 physicians’ interviews denote that PIs or APs experienced such a conflict. Moreover, one PI expressed off record the opposite view. He does not experience such a conflict because, according to him, even patients allocated to the placebo arm benefit from the improved care provided by RCTs compared to routine care. In contrast, as noted previously, four CRAs expressed the feeling that it might be disappointing for patients to be allocated to the placebo arm (S4 Table). However, beside this expression of empathy, none of the CRAs’ interviews explicitly revealed an internal conflict. One must take into account, however, that CRAs are not involved in clinical care; their role is exclusively related to research. The explicit expression of an ethical concern would be most unusual in the context of these interviews conducted at their workplace as it would represent a conflict of loyalty.

### A personal memory of healing unexplained by medicine

All 30 interviewees were asked to narrate a personal memory of medically unexplained healing. As many health professionals often started their answer in general terms about medical stories, the interviewer insisted by asking them about a story that involved the interviewee in person. Physicians answered this question rather hesitantly and with long pauses. In the content analysis we kept track of who was said to benefit from the unexplained healing (see all quotes in S12 Table). We considered two classes of beneficiaries: children, or adults described with childish characteristics by the interviewee, on the one hand and adults on the other. As an example of the first class, PI-5 recounted: *"When I was a child I had a lot of difficulties sleeping*. *Sometimes*, *my mother gave me sweetened water while saying that it was a medication*. *It worked and I have done the same with my children*.*"* A typical example of the second class was given by patient P-1, who recounted: *"We have a friend who has had several cancers*, *four or five*. *He has had a brain surgery*, *a lot of treatment and he is still there*. *His wife got leukemia and died*, *but her husband is doing great*. *It's just like a miracle"*. This sorting was performed for three categories of interviewees: patients, CRA and physicians (either PI or AP). The answer of one PI was not taken into account because he did not narrate a relevant story despite the interviewer's insistence. Most physicians (eight of 11) evoked a memory where the beneficiary was a child (6 cases) or an adult with childlike characteristics (2 cases). In contrast, in all but two patients' stories (10 of 12), the beneficiary was an adult. Likewise, four out of six CRA evoked an adult as a beneficiary of the unexplained healing (Table 7).

## Comments

Our observations are consistent with previous studies reviewed by Bishop et al. (2012) showing that most patients participating in RCTs do not understand the scientific need for placebo treatment [21]. Findings from other studies not reviewed by Bishop et al. (2012), supported the same view [28, 29]. Contradictory findings have also been reported. In one study (also not reviewed by Bishop et al.) of the interviews of 50 patients suffering from Parkinson’s disease and involved in RCTs, the patients seemed to have a good understanding of a placebo-controlled trial [30]. These patients, however, were interviewed by means of a standardized questionnaire that did not explicitly probe this understanding. Moreover, all patients were included in the placebo arm and interviewed after allocation disclosure. These conditions might explain their better understanding.

Thus, in line with the literature, our observations cast doubts concerning the effectiveness of the procedures that are brought into play to ensure the informed consent of the patient. In particular, although all patients had signed a consent form stating they might be allocated to a placebo treatment explicitly described as inactive, half of them did not realized that they might actually receive a sugar pill. It is true that these consent forms used the wordings “placebo treatment”, “dummy treatment” and “inactive substance” but not the more explicit one “sugar pill”. In this respect these French consent forms were similar to those used in Spain, Finland and the UK: placebo treatment is rarely described as a sugar pill [31–33]. In contrast, in a study about the effects of open-label placebo, placebo pills were explicitly described as “made of an inert substance like sugar pills” [34]. Following this last study, Blease et al. suggested that open-label placebo prescription would be ethically acceptable as long as ambiguities in the disclosure are eliminated as much as possible [35]. Thus, RCT consent forms should describe placebo treatment using most explicit wordings such as “sugar pill”.

This weakness in the consent forms should be corrected but it cannot explain by itself why many patients do not understand what a placebo treatment is. Indeed, it is likely that several patients did not read the consent form before signing it [36]. All of our observations point in another direction. Indeed, seven of eight PIs explicitly said that they select which patients will be asked to participate in an RCT. They justified this by the need to select patients who will be compliant with the treatment. This bias in participant recruitment has been reported previously: one of the nine PIs interviewed by Lawton et al. (2012) explicitly said that he and coworkers do not ask “people [who] are not really going to stay the course” [27]. In other studies about RCT recruitment PIs expressed their difficulties to recruit enough RCT participants, but did not evoke such a selection process [24, 26]. In the present study, although the criteria of this selective recruitment appear as rather subjective, they are consistent between PIs. PIs select patients who do not ask too many questions, those with a personality that is not too strong while being positive. These types of criteria have been termed by others "dispositional optimism" [14–16] and "agreeableness" [17, 18]. Consistently, all PIs believed that they exerted a strong influence on patients' decision to participate in an RCT.

That none of the 12 physicians expressed a conflict between their clinical and research roles may seem at odds with previous studies [22–25]. It must be acknowledged, however, that we did not specifically question them on this issue. Moreover, that our interviews were conducted before unveiling treatment allocation might have also contributed to this apparent lack of conflict. Indeed, other authors reported that emotional conflicts particularly come to the fore when professionals notify patients about their treatment allocation [25]. Furthermore, Easter and coworkers (2006) reported that, although several physicians involved in RCTs viewed care and research as conflicting activities, others disagreed and most RCT participants stated that researchers provide a better care than standard healthcare providers [23]. In our study one PI expressed the same opinion off record and this is likely to be shared by others PIs.

When asked to explain the placebo response, most PIs, APs and CRAs pointed to the role of the interrelationship with health professionals. The importance of this interrelationship has been repeatedly demonstrated [8, 12]. However, most previous studies only investigated the interrelationship between a single clinician and her patient. Here we investigated the distinct roles of PIs, APs and CRAs with patients involved in RCTs and our observations suggest that all three types of health professionals play a role. However, to our surprise, the interviewees' opinions suggest that the most influential health professionals are not the APs. Indeed, while most CRAs and most PIs believe that they may exert an influence on the placebo response, APs do not think so. In agreement with APs' opinions, their patients believed that their treatment response was not influenced by APs. This is all the more surprising in that APs frequently met their patients during the course of the RCT whereas PIs usually met them only once at the time of inclusion. Therefore, we observed an ambivalent representation of the placebo response, at least among the physicians that we interviewed. These physicians acknowledged that, in general, the interrelationship between health professionals and patients plays a role in the placebo response, but they were not ready to admit that they are personally involved in the placebo response of a specific patient.

However, it is also possible that the interrelationship between APs and their patients was really weaker than expected from studies about the interrelationship between outpatients and their physicians. Indeed, patients involved in RCTs do not select their physician whereas outpatients involved in ordinary clinical care do so. In RCTs, patients’ and APs’ feelings that their relationship has been imposed on them by others might contribute to weakening it. In contrast, the interrelationship between a PI and a patient might be stronger than anticipated because the PI selects the patient and because the patient gives him/her his consent on the basis of his feelings and trust rather than on the basis of rational arguments. Therefore, the formally free consent given by the patient, as well as his/her feeling that he/she has been chosen by the PI, might reinforce the relationship between patients and PIs. Moreover, this PI—patient interrelationship might be especially strong in patients’ imagination because of the aura that PIs may have. Indeed, all but one PI were directors of their department. Finally, CRAs' opinions suggest that they might have an influence on the treatment response. Altogether, the placebo response in the context of RCTs appears much more complex than in the context of a one-to-one dialogue between a physician and his/her outpatient. Moreover, our study on placebo representations further supports the classical view of the placebo effect. Accordingly and with reference to the etymologic meaning of the word "placebo" (*I will please*), many researchers in the field have expressed the view that the meaning reflects a reality, *i*.*e*., the size of the placebo response depends on the strength of an interrelationship in which patients and health professionals do their best to please each other [3]. However, this type of interrelationship produces effects only as long as all the partners stick to their complementary roles. Our observations suggest that this is actually the case.

Several lines of observation suggest that many RCT participants were in a childlike status. First, according to PIs, their decision to participate in an RCT was easily influenced. Second, CRAs believed they influence their placebo response through the “maternal” type of care they provided. Third, the sex distribution between PIs and CRAs was in line with the view that they played a paternal and a maternal role, respectively. Fourth, this sex distribution was in line with corresponding differences in the conceptualization of the placebo response. Indeed, while 11 out of 12 physicians put forward a neurobiological explanation, only one CRA did while the five others suggested a psychological interpretation instead. However, we do not infer from our observations that health professionals deliberately choose a paternalistic attitude towards RCT participants. Our observations are in line with a study reporting that half the patients did not assess the benefits or risks when they consented to participate in a RCT because they trusted their physician to know what is the best for them [37]. Corrigan (2003) and Levy (2014) question an idealistic view of informed consent when it is considered as “an ethical panacea to counter paternalistic medical practices” [38, 39]. They advocate for a more realistic view about informed consent that should take into account the social processes involved when patients consent to take part in RCTs.

Our interpretation regarding the complementary roles of health professionals and patients involved in RCTs is in line with that expressed by Miller, Colloca and Kaptchuk (2009) about the placebo response. They stated (p.12): "As social animals we are attuned from infancy to look to authoritative or protective figures—initially, our parents—to intervene to relieve distress… From a psychodynamic perspective, the healer’s authority and ability to comfort may be a projection of parental care, operating by a process of transference. Both conditioning from prior exposures to healers and expectations, as well as anxiety reduction, generated by the healer are likely to activate the placebo effect" [40]. Accordingly, the memories narrated by physicians about an instance of medically unexplained healings suggest that they were conscious, in a certain way, that the physician-patient relationship involves emotional components related to parental care. However, their reluctance to narrate a memory that involved them in person suggests that they prefer to ignore this subjective knowledge.

Most patients did not consider themselves easily influenced. This might seem inconsistent with the fact that half the patients did not realize that a placebo treatment is a sham treatment. However, their narratives about an instance of unexplained healing suggest that most of them were not conscious of their projections on health professionals. Moreover, an empirical study had already pointed out this contradiction [34]. Patients suffering from irritable bowel syndrome were randomized to two groups. One group openly received a placebo medication explicitly described as "a placebo pill made of an inert substance" (page 1). This prescription went with a comment stating that similar placebo treatments "have been shown in clinical studies to produce significant improvement through mind-body self-healing processes" (page 1). The control group received no treatment and the same quality of interaction with health professionals. The authors observed that the open-label prescription of a placebo produced significantly greater improvement than no treatment. They concluded that placebo prescription without deception might be an effective treatment [34]. However, when commenting on this study, Robert Ader suggested another interpretation. He noted that the patients received two conflicting messages: on the one hand they were informed that they would be receiving an inert pill, but on the other they were told that such a prescription had been shown to produce significant improvement. Because the same clinician delivered both messages and because patients are sufferers seeking medical help, the second message was more meaningful and persuasive to them than the first [41]. Our observations accord with this alternative interpretation. Patients selected for RCTs have a high level of confidence in their PI and it seems very difficult for most of them to realize that they might receive a sugar pill. Moreover, we observed that the explicit expression "sugar pill' to portray a placebo medication was never used by our sample of health professionals. They often used the expression "placebo treatment" or "inactive treatment", thus, feeding patients' false belief that in any case they were being treated.

Placebo-controlled RCTs are required to assess the effectiveness of new treatments. However, their relevance is based on the assumption that the patients involved in RCTs reflect the general population. Our study suggests that this is not the case. Patients are often selected on the basis of their character traits. Previous studies reported that patients with these traits (dispositional optimism, agreeableness) showed a larger placebo response than others [14–18] at least in some situations [13, 19]. It might be argued that the placebo component inherent in patients' responses to active treatment is also enhanced in this selected population and that the difference in improvement between the active treatment and the placebo one remains the same. However, this assumption has not been tested. Moreover, one could oppose that this selection of patients might also decrease the variability of the placebo response, thus increasing the likelihood that a modestly active treatment could be asserted to induce a statistically significant improvement. Finally, the results reported in RCTs might differ even more widely from those observed in the general population regarding psychotropic drugs prescribed either for mental disorders of for psychiatric comorbidities associated with somatic diseases. Therefore, our study calls for taking more into account the subjective and unconscious incentives that play a role in RCTs and may be sources of biases in their conclusions.

## Limitations

For ethical reasons and in order to avoid biases caused by unconscious justifications, RCT patients and their health professionals were interviewed after the end of the RCTs they participated in, but before the blinded allocation to placebo or active treatment had been unveiled. However, we did not attempt to link, after interviews, opinions stated by patients and their specific AP to the treatment allocation. Indeed, this study was performed in the context of multicenter RCTs initiated and supported by pharmaceutical companies that were reluctant to give us the breaking code.

Another limitation relates to the use of semi-structured interviews, which strongly limits the number of subjects. Our study was conducted in the context of classical Phase-3 RCTs involving PIs, APs and CRAs. Generalization of our views to other types of RCTs or to other clinical situations involving a placebo treatment might require further investigations. Finally, this study has been performed in the context of two neurodegenerative diseases and generalization to RCTs in other medical contexts should be cautiously considered.

## Conclusion

According to ethical codes about informed consent to participate in RCTs, informed patients and health professionals are equal partners sharing the same information and the same aim: to advance medical knowledge. However, in line with previous studies [42], we observed a large gap between these ethical codes and reality. Moreover, we believe that it is illusory to try to fill this gap by only reinforcing formal procedures regarding informed consent. Indeed, owing to patients' selection and to parental-like care, the interrelationship between health professionals and patients involved in RCTs appears largely asymmetrical. In order to rebalance the relationship between patients and physicians, ethicists advocate for physician training aimed at improving their patient-centered communication skills and empathy [7, 39]. However, we agree with Blease (2012) that “communication is not a tool that the doctor uses like a drug” [7]. Although physician empathy is critical for patient health, there is no strong support for the view that it is teachable through medical education [43–45]. Moreover, empathy, defined as the ability to share the emotions of others, intrinsically includes affective aspects that seem more difficult to change in an educational setting [45]. To overcome this difficulty several authors have offered medical students narrative exercises that involve their personal experience of illness [46–48]. This reflexive approach might also be relevant for the training of physicians involved in RCTs. Indeed, our observations suggest that, when adequately queried, health professionals might be able to draw from their personal experience a sensitive knowledge of what patients experience when involved in RCTs.

## Supporting Information

S1 Table Opinion 3a: In RCTs, placebo is a methodological requirement to assert the effectiveness of the new treatment under investigation. (DOCX)

S2 Table Opinion 3b: How does placebo work: neurobiological effect, belief/expectation or both? (DOCX)

S3 Table Opinion 3c: The interrelationship with health professionals is involved. (DOCX)

S4 Table Opinion 3d: Patients allocated to placebo might feel disappointed. (DOCX)

S5 Table Opinion 4a: The PI has subjective criteria for patients’ inclusion. (DOCX)

S6 Table Opinion 4b: The PI also considers the family circle of the patient. (DOCX)

S7 Table Opinion 4c: The PI acknowledges that he influences the patient’s decision. (DOCX)

S8 Table Opinions 5a and 5b: Do you think you have an influence on the placebo response? (DOCX)

S9 Table Opinion 6a: As an AP, I think that I had an influence on the treatment response of my patient. (DOCX)

S10 Table Opinion 6b: I think that my physician (i.e. my AP) had an influence on my treatment response. (DOCX)

S11 Table Opinion 6c: I think that APs might influence the placebo response. (DOCX)

S12 Table Opinion 7: Who is said to benefit from the unexplained healing? (DOCX)
