# Supplementary material for: How Health Professionals Conceptualize and Represent Placebo Treatment in Clinical Trials and How Their Patients Understand It: Impact on Validity of Informed Consent
Source: PLoS One. 2016 May 19;11(5):e0155940. doi: 10.1371/journal.pone.0155940 (PMC4873029; doi:10.1371/journal.pone.0155940)
Supplement: S1 Table — (DOCX) [file pone.0155940.s001.docx]

**Table S1.** Opinion 3a: In RCTs, placebo is a methodological requirement to assert the effectiveness of a new treatment under investigation

| **Principal investigators** | | | |
| --- | --- | --- | --- |
| PI-1 | "It is a mandatory methodological basis to prove the effectiveness of a medication." | | |
| PI-2 | "It prevents the doctor from being maneuvered… to overrate or underrate certain symptoms in a conscious or unconscious way." | | |
| PI-3 | "It is the research gold standard … to avoid biased selection and evaluation." | | |
| PI-4 | "It is mandatory…the only acceptable proof within the evidence-based medicine framework." | | |
| PI-5 | "There is nothing better from the methodological point of view to demonstrate the effectiveness of a drug treatment." | | |
| PI-6 | "It is the most efficient way to get the least subjective conclusion… but it is difficult to achieve for many pathologies." | | |
| PI-7 | "It is what appears to me today as the soundest method to avoid overestimating the treatment effect." | | |
| PI-8 | "A comparative trial is mandatory. When there is no proven therapeutic, it is usually a placebo….This is evidence-based medicine." | | |
| **Associated physicians** | | | |
| AP-1 | | "...it is required, owing to the large size of the placebo effect regarding Parkinson's disease." | |
| AP-2 | | "…to evaluate the effectiveness of a drug compared to a control." | |
| AP-3 | | "to avoid any bias in the interpretation of the effectiveness of the treatment." | |
| AP-4 | | "I think that it is the best way to assess the effectiveness of a product." | |
| **Clinical research associates** | | | |
| CRA-1 | | | "It is one way of proving the effectiveness of a treatment… it is very common." |
| CRA-2 | | | "It seems to me barely escapable if we want to prove the effectiveness of a treatment." |
| CRA-3 | | | "It can be good as long as the placebo is the only way to compare …when reference treatment doesn’t exist." |
| CRA-4 | | | "If one could know which treatment patients have received, one could influence the size of the response." |
| CRA-5 | | | "This is the way to go to obtain an unbiased opinion about the effectiveness of a treatment… as long as it is regulated by the law, I think that it is essential." |
| CRA-6 | | | "…in order to test the effectiveness of a drug we have to perform studies with placebo." |
| **Patients** | | | |
| Pat-1 | | *Did not mention the methodological requirement for a controlled RCT despite insistence.* | |
| Pat-2 | | *Vague mentioning of a need, but without understanding the usefulness of placebo control:* "It is essential for launching a new drug." | |
| Pat-3 | | *Did not mention the methodological requirement for a controlled RCT despite insistence.* | |
| Pat-4 | | *Did not mention the methodological requirement for a controlled RCT despite insistence.* | |
| Pat-5 | | "…to know for sure if the drug is working or not." | |
| Pat-6 | | "…to be able to check whether the drug is active or not." | |
| Pat-7 | | *Did not mention the methodological requirement for a controlled RCT despite insistence.* | |
| Pat-8 | | *Did not mention the methodological requirement for a controlled RCT despite insistence.* | |
| Pat-9 | | *Did not mention the methodological requirement for a controlled RCT despite insistence.* | |
| Pat-10 | | *Did not mention the methodological requirement for a controlled RCT despite insistence.* | |
| Pat-11 | | *Vague mentioning of a need, but without understanding the usefulness in RCTs:* "It is essential for research studies*,* but for the patient…" | |
| Pat-12 | | *Did not mention the methodological requirement for a controlled RCT despite insistence.* | |
