# Supplementary material for: How Health Professionals Conceptualize and Represent Placebo Treatment in Clinical Trials and How Their Patients Understand It: Impact on Validity of Informed Consent
Source: PLoS One. 2016 May 19;11(5):e0155940. doi: 10.1371/journal.pone.0155940 (PMC4873029; doi:10.1371/journal.pone.0155940)
Supplement: S2 Table — (DOCX) [file pone.0155940.s002.docx]

**Table S2**. Opinion 3b: How does placebo work: neurobiological effect, belief/expectation or both?

| **Principal Investigators** | | |
| --- | --- | --- |
| PI-1 | "For me it is the expectation… the brain… the nervous system and the body will respond to this expectation." | |
| PI-2 | "…an endorphin release, the neurotransmitter Y or Z that improves psyche." | |
| PI-3 | "…by neurotransmitters… the belief will result in endorphin secretion." | |
| PI-4 | "The one who thinks he received the active molecule… our brain or our psyche is able to secrete a certain number of neurotransmitters, hormones…" | |
| PI-5 | "Suggestibility has a very biological effect… it can result in the release of neurotransmitters." | |
| PI-6 | "…believing in it… makes biochemical modifications… the neurotransmitters…" | |
| PI-7 | "The placebo works through very physiological mechanisms…dopamine release…" | |
| PI-8 | "When people have an expectation…at the cerebral level…neuronal networks might be affected, but we are still awaiting a full scientific understanding." | |
| **Associated physician** | | |
| AP-1 | "There is a neurophysiological explanation… structures related to mechanisms of autosuggestion…" | |
| AP-2 | "…the placebo activates the reward system… the mesocorticolimbic system…" | |
| AP-3 | "It is a psychological context that may influence the neurotransmitters…" | |
| AP-4 | "There is a double suggestion… it depends if we believe in it or not." | |
| **Clinical research associates** | | |
| CRA-1 | | "It is very psychological… it depends on patients…their way of believing in it." |
| CRA-2 | | "… moreover, it affects the way people feel." |
| CRA-3 | | "…because the patient thinks "well I am gonna get something" so he will inevitably get better." |
| CRA-4 | | "For some patients just taking something, even if it is a placebo, will have an effect...They must be thinking…" |
| CRA-5 | | "…when we believe in…when we feel good, there are some mechanisms at the level of the neurotransmitters that act...." |
| CRA-6 | | "It is psychosomatic; there are patients who, just by thinking that they are getting a drug, can cure themselves." |
