# Supplementary material for: How Health Professionals Conceptualize and Represent Placebo Treatment in Clinical Trials and How Their Patients Understand It: Impact on Validity of Informed Consent
Source: PLoS One. 2016 May 19;11(5):e0155940. doi: 10.1371/journal.pone.0155940 (PMC4873029; doi:10.1371/journal.pone.0155940)
Supplement: S3 Table — (DOCX) [file pone.0155940.s003.docx]

**Table S3**. Opinion 3c: The interrelationship with health professionals is involved

| **Principal Investigators** | | |
| --- | --- | --- |
| PI-1 | "There is also the effect of medical management… a different relationship… it is the patient being at the focal point…" | |
| PI-2 | "…the feeling that the person has received more global and personalized medical care." | |
| PI-3 | [the placebo effect] "I do not see it as something personal but linked to the team." | |
| PI-4 | *Not mentioned* | |
| PI-5 | "…the result of an interaction between a patient, a doctor and a context of a therapeutic trial." | |
| PI-6 | "The placebo action … it goes through the patient – doctor relationship." | |
| PI-7 | *Not mentioned* | |
| PI-8 | "This is related to the physician – patient relationship…physician's persuasion, empathy…When a patient comes here he expects an improvement of his symptoms." | |
| **Associated physician** | | |
| AP-1 | "The medical management has a beneficial effect." | |
| AP-2 | "The interaction patient – physician is essential" | |
| AP-3 | *Not mentioned* | |
| AP-4 | "Our way of explaining things may have an effect on the patient's health." | |
| **Clinical research associates** | | |
| CRA-1 | | "Patients get better because their follow-up is more frequent, it's real medical management." |
| CRA-2 | | "…because the patients are taken care of… they are warmly supported, listened to, that's all." |
| CRA-3 | | "It is different medical management [as compared to a usual consultation]… it is whole management." |
| CRA-4 | | "It is a feeling of being taken care of, managed and thus, they feel better." |
| CRA-5 | | *Not mentioned* |
| CRA-6 | | *Not mentioned* |
