# Supplementary material for: How Health Professionals Conceptualize and Represent Placebo Treatment in Clinical Trials and How Their Patients Understand It: Impact on Validity of Informed Consent
Source: PLoS One. 2016 May 19;11(5):e0155940. doi: 10.1371/journal.pone.0155940 (PMC4873029; doi:10.1371/journal.pone.0155940)
Supplement: S4 Table — (DOCX) [file pone.0155940.s004.docx]

**Table S4**. Opinion 3d: Patients allocated to placebo might feel disappointed

| **Principal Investigators** | | |
| --- | --- | --- |
| PI-1 | *Not mentioned* | |
| PI-2 | *Not mentioned* | |
| PI-3 | *Not mentioned* | |
| PI-4 | *Not mentioned* | |
| PI-5 | *Not mentioned* | |
| PI-6 | *Not mentioned* | |
| PI-7 | *Not mentioned* | |
| PI-8 | *Not mentioned* | |
| **Associated physicians** | | |
| AP-1 | *Not mentioned* | |
| AP-2 | "There is an ethical concern… giving a placebo to patients could mean not giving them the chance to be treated." | |
| AP-3 | *Not mentioned* | |
| AP-4 | *Not mentioned* | |
| **Clinical Research associates** | | |
| CRA-1 | | "For patients, an RCT using a proven medication is more ethical." |
| CRA-2 | | "…as long as it does not harm patients… I, if I were a patient, I would like to get the treatment." |
| CRA-3 | | *Not mentioned* |
| CRA-4 | | "…it's true it's unlucky for the one receiving the placebo." |
| CRA-5 | | *Not mentioned* |
| CRA-6 | | "It's true that patients don't really like to know they’re only getting the placebo." |
| **Patients** | | |
| Pat-1 | *Not mentioned* | |
| Pat-2 | *Not mentioned* | |
| Pat-3 | "If for six months we eat a placebo, we will feel more like a guinea-pig than anything else." | |
| Pat-4 | *Not mentioned* | |
| Pat-5 | *Not mentioned* | |
| Pat-6 | *Not mentioned* | |
| Pat-7 | *Not mentioned* | |
| Pat-8 | *Not mentioned* | |
| Pat-9 | *Not mentioned* | |
| Pat-10 | *Not mentioned* | |
| Pat-11 | "It is a pity for those who get the placebo… that's half the patients losing..." | |
| Pat-12 | *Not mentioned* | |
