# Supplementary material for: How Health Professionals Conceptualize and Represent Placebo Treatment in Clinical Trials and How Their Patients Understand It: Impact on Validity of Informed Consent
Source: PLoS One. 2016 May 19;11(5):e0155940. doi: 10.1371/journal.pone.0155940 (PMC4873029; doi:10.1371/journal.pone.0155940)
Supplement: S5 Table — (DOCX) [file pone.0155940.s005.docx]

**Table S5:** Opinion 4a: The PI has subjective criteria for patients’ inclusion

| **Principal Investigators** | |
| --- | --- |
| PI-1 | "…intuitive criteria… anxious patients who have very negative representations of the drugs, those ones I do not ask them. You will sluggishly describe the RCT to suspicious patients hoping unwittingly that you did it sluggishly enough so that he does not take part in it, but still having done your job." |
| PI-2 | "We prioritize patients we have known for a long time, the ones we know we can rely on." |
| PI-3 | "…a patient who looks like a good guy for an RCT… a compliant patient... We wish somehow that the patient's motivation will be higher than that just required to sign up for the protocol…We would not ask patients with a schoolteacher profile. These people systematically question what physicians say, they systematically look elsewhere for pieces of information and they almost never do what we ask them to do." |
| PI-4 | "I would say we mainly ask people… without much personality… we are mostly looking for people with a compliant profile… People who are likely to protest, or to complain… we often have trouble with them,…moaning about so-called side effects." |
| PI-5 | "What is going to be important is the ability of the patient to accept the constraints related to the study… if I see he is not reliable… The study prevails…in contrast to the medical act which is totally up to the doctor." |
| PI-6 | *No subjective criteria*: "I ask almost all my patients". |
| PI-7 | "Many patients do it their own way… The patient not following what I advise him to do… I can hardly ask him." |
| PI-8 | "I ask everybody, but when we know the answer. For example the patient, who just left, told me "I am anti medication". Of course, I will not ask him to take part in an RCT." |
