# Supplementary material for: How Health Professionals Conceptualize and Represent Placebo Treatment in Clinical Trials and How Their Patients Understand It: Impact on Validity of Informed Consent
Source: PLoS One. 2016 May 19;11(5):e0155940. doi: 10.1371/journal.pone.0155940 (PMC4873029; doi:10.1371/journal.pone.0155940)
Supplement: S6 Table — (DOCX) [file pone.0155940.s006.docx]

**Table S6.** Opinion 4b: The PI also considers the family circle of the patient

| **Principal investigators** | |
| --- | --- |
| PI-1 | "It's a feeling … for example a patient under a mistrustful spouse's thumb." |
| PI-2 | "…a patient supported by a family circle." |
| PI-3 | "Many relationships play a role…spouse… children… parents… patients' associations." |
| PI-4 | *Not mentioned* |
| PI-5 | "The patient needs to have people around him with a positive attitude towards treatment." |
| PI-6 | *Not mentioned* |
| PI-7 | *Not mentioned* |
| PI-8 | *Not mentioned* |
