# Supplementary material for: How Health Professionals Conceptualize and Represent Placebo Treatment in Clinical Trials and How Their Patients Understand It: Impact on Validity of Informed Consent
Source: PLoS One. 2016 May 19;11(5):e0155940. doi: 10.1371/journal.pone.0155940 (PMC4873029; doi:10.1371/journal.pone.0155940)
Supplement: S7 Table — (DOCX) [file pone.0155940.s007.docx]

**Table S7.** Opinion 4c: The PI acknowledges that he influences the patient’s decision

| **Principal investigators** | |
| --- | --- |
| PI-1 | "You play a huge role in the patient's decision up to a patient following blindly what you say. This happens frequently." |
| PI-2 | "Huge role… Either I tell them about it [and they accept], or I don't." |
| PI-3 | "I believe we play a huge role." |
| PI-4 | "The PI's representation is really important and inevitably influences the patient's decision." |
| PI-5 | "We strongly influence the patient's decision… If I set my mind on getting someone to take part, he will take part." |
| PI-6 | "PIs hugely contribute to a patient 's consent." |
| PI-7 | "It seems obvious to me that the way the PI presents the study…the PI will get the patient's consent. He [the patient] completely relies on what the doctor tells him." |
| PI-8 | "I try to be as neutral as possible. The truth is that we are never fully neutral. I never make them do it…but, unconsciously, when I believe that this new treatment might be really effective for this patient, I say: "Be aware that there is no alternative, it might be an opportunity to take it." |
