# Supplementary material for: How Health Professionals Conceptualize and Represent Placebo Treatment in Clinical Trials and How Their Patients Understand It: Impact on Validity of Informed Consent
Source: PLoS One. 2016 May 19;11(5):e0155940. doi: 10.1371/journal.pone.0155940 (PMC4873029; doi:10.1371/journal.pone.0155940)
Supplement: S8 Table — (DOCX) [file pone.0155940.s008.docx]

**Table S8.**Opinions 5a and 5b: Do you think you have an influence on the placebo response?

| **Principal investigators** | | |
| --- | --- | --- |
| PI-1 | "Yes,… our enthusiasm, our belief in the value of this new drug, plays a major role on the patient's involvement… the expectation will be stronger." | |
| PI-2 | "I don't know, I hope I don't have much influence on this placebo effect…Of course there is the environment, the team…" | |
| PI-3 | "I don't know [in the context of an RCT], I don't see it as something personal, but rather related to the team." | |
| PI-4 | "Yes, maybe. The placebo effect results more from the patient than from the doctor, even if the doctor may contribute to it through persuasion." | |
| PI-5 | "If we are really influential, we might exert a positive effect of the treatment, but if the patient is in the placebo arm, as a result, the placebo will have a larger effect." | |
| PI-6 | "I think that it [the placebo effect] takes place right from the beginning when we present the new molecule, and thus it results from the hope the patient has in the new molecule." | |
| PI-7 | "I am so enthusiastic that, maybe, I shouldn't talk to patients to get their consent…because my enthusiasm will be passed on to the patient." | |
| PI-8 | "No doubt that I induce a placebo effect… When we are a principal investigator we bring a certain enthusiasm and patients may feel it. Yes, I believe we do exert an influence." | |
| **Clinical Research Associates** | | |
| CRA-1 | | "Yes, we exert a huge influence…It is a little bit like a maternal attitude, because as soon as they have a concern, they call me. Some patients say: "we feel pampered, like with a mom." |
| CRA-2 | | "Yes, I think so…We have a privileged relationship with patients, we know them well, they call us, we call them. We pamper them a little bit." |
| CRA-3 | | *Maybe:* "I don't know whether I take part in the placebo effect, but…The fact that we take good care of patients, that we see to patients every needs. Obviously, a relationship is taking place." |
| CRA-4 | | *Maybe:* "I don't know… but I think trustworthy relationships are progressively occurring. It's more like it…. We often spend more time with them than the doctor…As soon as they join the study, they no longer call the doctor, they call us all the time." |
| CRA-5 | | "Yes, we emphasize the belief, the belief of the doctor representing the study…It has maybe something to do with the belief I can have when I describe the study to a patient." |
| CRA-6 | | *Maybe:* "I really try to keep a neutral attitude…I try to be… to be close, but not too close to the patient." |
