# Supplementary material for: How Health Professionals Conceptualize and Represent Placebo Treatment in Clinical Trials and How Their Patients Understand It: Impact on Validity of Informed Consent
Source: PLoS One. 2016 May 19;11(5):e0155940. doi: 10.1371/journal.pone.0155940 (PMC4873029; doi:10.1371/journal.pone.0155940)
Supplement: S9 Table — (DOCX) [file pone.0155940.s009.docx]

**Table S9.** Opinion 6a: As AP, I think that I had an influence on the treatment response of my patient

| **Associated physician** | |
| --- | --- |
| AP-1 | "I think my behavior did not affect my patient's improvement." |
| AP-1' | "I feel I have nothing to do with the fact that it [the treatment] had no effect on this patient." |
| AP-2 | "I hardly play a role in it. It is mainly global medical care." |
| AP-2' | *The AP dodges the issue regarding this patient, who dropped out because of side effects.* |
| AP-2'' | *The AP dodges the issue regarding this patient who seems to feel better.* |
| AP-2''' | "I don't think I have anything to do with it. I keep a neutral attitude." *(improved patient)* |
| AP-3 | *The AP dodges the issue because she only met the patient once.* |
| AP-4 | *The AP dodges the issue.* |
