# Supplementary material for: How Health Professionals Conceptualize and Represent Placebo Treatment in Clinical Trials and How Their Patients Understand It: Impact on Validity of Informed Consent
Source: PLoS One. 2016 May 19;11(5):e0155940. doi: 10.1371/journal.pone.0155940 (PMC4873029; doi:10.1371/journal.pone.0155940)
Supplement: S10 Table — (DOCX) [file pone.0155940.s010.docx]

**Table S10.** Opinion 6b: I think that my physician (*i.e.* my AP) had an influence on my treatment response

| **Patients** | |
| --- | --- |
| Pat-1 | "Yes. At the beginning, I was so happy to take part in the study that I was thinking: "that's it everything is gonna get better. I felt I was trembling less." |
| Pat-2 | "I don't think that he [the PA] played any role in how I felt. " |
| Pat-3 | "No I don't think so… The doctor didn't influence it [the course of my disease]." |
| Pat-4 | *No opinion* |
| Pat-5 | "No, I can't see what influence he may have." |
| Pat-6 | " No, my personal relationship with my doctor was not significant enough… so I can't think of it." |
| Pat-7 | "No, I don't think so. He did a good job, he was nice and a good listener, but I do not see why…no. " |
| Pat-8 | "No, really, I don't think so." |
| Pat-9 | "Oh! Doctors...! "(huge sigh) |
| Pat-10 | *No opinion* |
| Pat-11 | "I can hardly think it will work for me [the placebo]." |
| Pat-12 | "No, he [his PA] does not influence me regarding my symptoms. " |
