# Supplementary material for: How Health Professionals Conceptualize and Represent Placebo Treatment in Clinical Trials and How Their Patients Understand It: Impact on Validity of Informed Consent
Source: PLoS One. 2016 May 19;11(5):e0155940. doi: 10.1371/journal.pone.0155940 (PMC4873029; doi:10.1371/journal.pone.0155940)
Supplement: S11 Table — (DOCX) [file pone.0155940.s011.docx]

**Table S11.** Opinion 6c: I think that APs might influence the placebo response

| **Clinical research associates** | |
| --- | --- |
| CRA-1 | "The way the doctor behaves during the consultation will inevitably have an influence." |
| CRA-2 | *No opinion* |
| CRA-3 | *No opinion* |
| CRA-4 | "Yes, some doctors are good listeners and will spend much more time than others. It might have an effect." |
| CRA-5 | "Yes. If he really believes in the molecule, he will present it in such a way the patient will believe in it too. If he is convinced, he will easily convince the patients." |
| CRA-6 | *No opinion* |
