# Supplementary material for: How Health Professionals Conceptualize and Represent Placebo Treatment in Clinical Trials and How Their Patients Understand It: Impact on Validity of Informed Consent
Source: PLoS One. 2016 May 19;11(5):e0155940. doi: 10.1371/journal.pone.0155940 (PMC4873029; doi:10.1371/journal.pone.0155940)
Supplement: S12 Table — (DOCX) [file pone.0155940.s012.docx]

**Table S12.** Opinion 7: Who is said to benefit from unexplained healing?

| **Principal investigators** | | |
| --- | --- | --- |
| PI-1 | [talking about an adult patient reacting really positively to placebo] "It might be, his youth, because he looked like a child. The way he trusted me, reminds me of the trust existing between a child and his parents for example… the softest existing placebo effect: a mum blowing gently on a wound." | |
| PI-2 | [talking about an adult patient reacting positively to placebo] "I remember a kid who had…" (Interviewer: "Was she a little girl? ") "No, a kid like, 30. I say *kid* because I know her parents." | |
| PI-3 | *An adult having headaches.* | |
| PI-4 | "One of my daughters…" | |
| PI-5 | "When I was a child I had a lot of difficulties sleeping. Sometimes, my mother gave me sweetened water while saying that it is a medication. It worked and I have done the same with my children." | |
| PI-6 | "I had a kid with strong migraine when he was young…" | |
| PI-7 | [talking about an adult patient reacting very positively to placebo] "I greatly admired him for what he was able to do, what he has done with such strength when treatments did nothing". (Off the record, this physician said that this patient reminded him of people from his childhood). | |
| PI-8 | "Regarding psychogenic disorders, in our society, most people who consult are women. I see too many of them. I cannot remember one in particular." | |
| **Associated physician** | | |
| AP-1 | "One of my sons…" | |
| AP-2 | "My poor father, who is sick… he needs to be stimulated… When my young niece comes, he smiles." | |
| AP-3 | "Healing? It is rather more with children… it is the magic water… " | |
| AP-4 | "It is a girl who felt faint, lapsed into unconsciousness… | |
| CRA | | |
| CRA-1 | | *An adult with a skin disease cured without any medical explanation.* |
| CRA-2 | | "My aunt told me that when she was a little girl she suffered from ear infection…" |
| CRA-3 | | *An adult being in a coma and who recovered despite a poor prognosis.* |
| CRA-4 | | *A close relative who recovered from a very serious disease.* |
| CRA-5 | | [about warts]. "I had them when I was a child… it happened in my grandparents' home." |
| CRA-6 | | [about a close relative who recovered from unexplained nauseas]. "She consulted a psychologist and he revealed problems during her childhood." |
| **Patients** | | |
| Pat-1 | "We have a friend who has had several cancers, four or five. He has had a brain surgery, a lot of treatment and he is still there. His wife got leukemia and died, but her husband is doing great. It's just like a miracle". | |
| Pat-2 | "My mother told me that I was very sick and that I nearly died, but I don't remember anything. I was too young. " | |
| Pat-3 | *An adult survived leukemia although he was sentenced to death by medicine.* | |
| Pat-4 | *The patient talks about his personal case.* | |
| Pat-5 | *A patient suffering from Parkinson's disease who recovered after a pilgrimage to Lourdes.* | |
| Pat-6 | *A patient suffering from severe multiple sclerosis during the day and presenting no symptoms at night.* | |
| Pat-7 | *A patient suffering from Parkinson's disease who recovered after a pilgrimage to Lourdes.* | |
| Pat-8 | *A patient suffering from Parkinson's disease who recovered after a pilgrimage to Lourdes.* | |
| Pat-9 | "My son, 4, put his hand on the stove of his grandmother… He was severely burnt… An old granny from the village "pulled out the fire"… he left her home laughing!" | |
| Pat-10 | *A woman, who could no longer walk, recovered after a pilgrimage to Lourdes.* | |
| Pat-11 | *In a movie a paralyzed person who walked again.* | |
| Pat-12 | *A patient who recovered after a pilgrimage to Lourdes.* | |
